# First Chromosomal Analysis in Hepsetidae (Actinopterygii, Characiformes): Insights into Relationship between African and Neotropical Fish Groups

**DOI:** 10.3389/fgene.2017.00203

**Published:** 2017-12-12

**Authors:** Pedro C. Carvalho, Ezequiel A. de Oliveira, Luiz A. C. Bertollo, Cassia F. Yano, Claudio Oliveira, Eva Decru, Oladele I. Jegede, Terumi Hatanaka, Thomas Liehr, Ahmed B. H. Al-Rikabi, Marcelo de B. Cioffi

**Affiliations:** ^1^Departamento de Genética e Evolução, Universidade Federal de São Carlos, São Carlos, Brazil; ^2^Secretaria de Estado de Educação de Mato Grosso (Seduc-MT), Cuiabá, Brazil; ^3^Departamento de Morfologia, Instituto de Biociências, Universidade Estadual Paulista, Botucatu, Brazil; ^4^Section Vertebrates, Ichthyology, Royal Museum for Central Africa, Tervuren, Belgium; ^5^Department of Fisheries and Aquaculture, Adamawa State University, Mubi, Nigeria; ^6^Institute of Human Genetics, University Hospital Jena, Jena, Germany

**Keywords:** fishes, molecular cytogenetics, chromosomal painting, comparative genomic hybridization (CGH), karyotype evolution

## Abstract

Hepsetidae is a small fish family with only the genus *Hepsetus*, with six described species distributed throughout the South, Central and Western regions of Africa, showing a close relationship with the Alestidae and some Neotropical fish families. However, no cytogenetic information is available for both Hepsetidae and Alestidae species, thus preventing any evolutionary comparative studies at the chromosomal level. In the present study, we are providing new cytogenetic data for *Hepsetus odoe*, including the standard karyotype, C-banding, repetitive DNAs mapping, comparative genomic hybridization (CGH) and whole chromosome painting (WCP), providing chromosomal patterns and subsidies for comparative cytogenetics with other characiform families. Both males and females *H. odoe* have 2n = 58 chromosomes (10m + 28sm + 20st/a), with most of the C-band positive heterochromatin localized in the centromeric and subtelomeric regions. Only one pair of chromosomes bears proximal 5S rDNA sites in the short arms, contrasting with the 18S rDNA sequences which are located in the terminal regions of four chromosome pairs. Clear interstitial hybridization signals are evidenced for the U1 and U2 snDNA probes, but in only one and two chromosome pairs, respectively. Microsatellite motifs are widely distributed in the karyotype, with exception for the (CGG)_10_, (GAA)_10_ and (GAG)_10_ probes, which highlight conspicuous interstitial signals on an unique pair of chromosomes. Comparative data from conventional and molecular cytogenetics, including CGH and WCP experiments, indicate that *H. odoe* and some Erythrinidae species, particularly *Erythrinus erythrinus*, share similar chromosomal sequences suggesting some relatedness among them, although bearing genomic specificities in view of their divergent evolutionary histories.

## Introduction

Characiformes comprises 24 families and more than 2100 species (Eschmeyer and Fong, 2017), distributed in many Neotropical and Ethiopian rivers (Nelson et al., 2016). As they are exclusively freshwater fishes, their evolutionary history is related with continents fragmentations and settlement and, with the development of natural barriers during their dispersion throughout secondary habitats (Vari and Malabarba, 1998; Oliveira et al., 2007).

The most primitive characiforms are the African citharinoids (Arroyave et al., 2013), and the relationship between the Neotropical and Ethiopian species may be closely linked with the Gondwana break-up, with a fast diversification established in a new habitat free of competition (Calcagnotto et al., 2005). Despite significant efforts on morphological and molecular analyzes, phylogenetic relationships are currently still uncertain for several groups and even the monophyly of Characiformes is still debated (Arcila et al., 2017).

The wide diversification of the characiforms is highlighted by the high karyotype variability found within distinct Neotropical groups, showing the fast evolution of these fishes as expected by the high fragmentation observed in the South American rivers, in contrast with the African ones, which presents lower fragmentation and variability (Ortí and Meyer, 1997; Oliveira et al., 2007). One example of such scenario concerns the Erythrinidae, a small family widely distributed throughout South America, consisting of the genus *Erythrinus*, *Hoplerythrinus*, and *Hoplias* (Oyakawa, 2003). Cytogenetics of the Erythrinidae fishes have been quite investigated over years, especially for *H. malabaricus* and *E. erythrinus*, where a variety of chromosomal features occurs even within a same nominal species, thus supporting the presence of species complexes (Bertollo, 2007; Cioffi et al., 2012). In fact, erythrinids hold a variety of different karyomorphs, with diploid numbers (2n) varying from 39 in *Hoplias malabaricus* (karyomorph D) to 2n = 54 in *Erythrinus erythrinus* (karyomorph A), in addition to distinct sex chromosomes systems with independent origins and particular evolutionary trajectories (Cioffi et al., 2013). The diploid number found for most *Erythrinus* species (2n = 54) is also the common one observed for Characiformes, which possibly represents the ancestral condition for this order (Oliveira et al., 2007; Cioffi et al., 2012). However, the full comprehension of the evolutionary relationships of its families is not clear until now. A recent phylogeny based on 1,051 genetic markers showed that both African Hepsetidae and Alestidae families have closer relationship and, in a lower scale, to other Neotropical families, such as Erythrinidae, Cynodontidae and Hemiodotidae, but not with Lebiasinidae and Ctenoluciidae (Arcila et al., 2017). This result is not fully consensual with some previous phylogenetic proposals (Ortí and Meyer, 1997; Buckup, 1998; Calcagnotto et al., 2005), where some of above families were found to be related.

Notwithstanding, except for the Erythrinidae (see above), most of these families remain with kayotypes poorly analyzed, thus limiting any evolutionary comparative studies among them at the chromosomal level. In this sense, karyological data for Hemiodontidae are mainly restricted to chromosome numbers although all species presenting the same diploid number (2n = 54) and bi-armed chromosomes (Arefjev, 1990; Porto et al., 1992, 1993; Arai, 2011). Concerning Cynodontidae, the only species analyzed up to now (*Rhaphiodon vulpinus*) also presented the same 2n and karyotype structure (Pastori et al., 2009). In turn, the available chromosome data for Lebiasinidae are also mainly restricted to chromosome numbers (Scheel, 1973; Arai, 2011), with exception for a few species (Arefjev, 1990; Oliveira et al., 1991). Despite such largely limitation, a high diversity characterizes their diploid numbers, which ranges from 2*n* = 22 in *Nannostomus unifasciatus*, to 2n = 46 in *N. trifasciatus* (Oliveira et al., 2007; Arai, 2011). Occasional occurrence of large metacentric pairs, such as in *N. unifasciatus* (Arefjev, 1990) points to Robertsonian fusions in the karyotype differentiation. *Pyrrhulina australis* and *Pyrrhulina* aff. *australis* share 2n = 40 (4st + 36a), however, a significant genomic divergence was found between them, evidencing that they correspond to distinct evolutionary units (Moraes et al., 2017). As regards to Ctenoluciidae, four species of the *Boulengerella* genus from the Amazon River basin (Brazil), showed 2n = 36 and a very similar karyotype organization. A conspicuous chromosomal heteromorphism in male specimens point to a possible XX/XY sex chromosome system in such species (de Souza E Sousa et al., 2017). Besides, *Ctenolucius hujeta* (2n = 36) is the only additional species of Ctenoluciidae that has its chromosomal number already analyzed (Arefjev, 1990), coinciding with those found for the *Boulengerella* species.

The Hepsetidae family contains only a single genus (*Hepsetus*) and, for a long time, *H. odoe* was considered the only valid species. However, five additional species have been described by recent studies: *H. kingsleyae*, *H. lineatus*, *H. occidentalis*, *H. cuvieri*, and *H. microlepis*, distributed throughout the South, Central and Western regions of Africa (Decru et al., 2012, 2013a,b, 2015), where they have great significance for local economy (Kareem et al., 2016). Despite the economic and evolutionary importance of this group, no chromosome data are available for any Hepsetidae species.

In the present study, we provide, for the first time, cytogenetic data for *Hepsetus odoe*, including the standard karyotype, C-banding, repetitive DNAs mapping, comparative genomic hybridization (CGH) and whole chromosome painting (WCP), in order to investigate its chromosomal patterns and provide subsidies for comparative analyzes with some Neotropical fish families. In this sense, this study represents the first one of a series focusing on the cytogenetics and cytogenomics of the African species toward their karyoevolutionary processes.

## Materials and Methods

### Specimens, Chromosome Preparations, C-banding and DNA Samples

**FIGURE 1:**
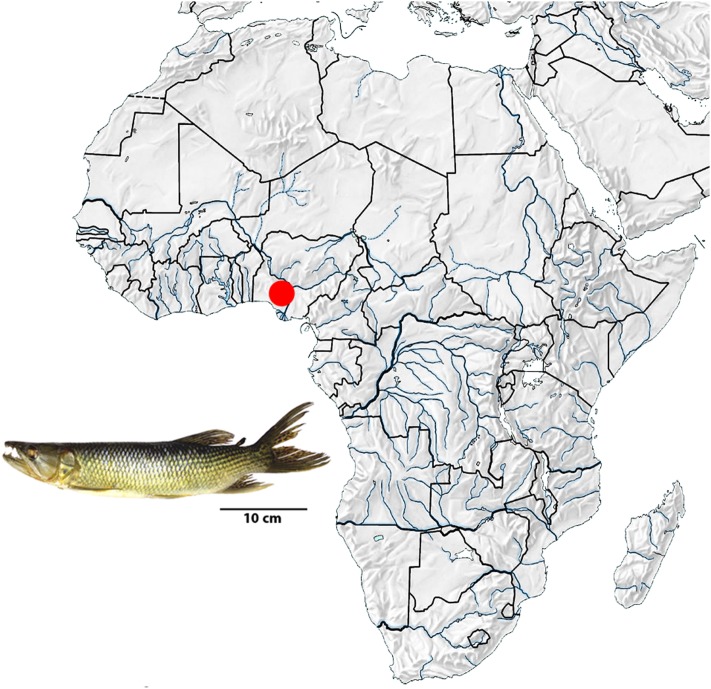
Map of Africa showing the collect location of the *Hepsetus odoe* specimens (red point) in Nigeria, Oluwa River, Niger River basin. Map of Africa was adapted from http://geografiahistoriajodar.blogspot.com.br/.

Eleven specimens of *Hepsetus odoe* (06 males and 05 females) from the Opa Reservoir, Obafemi Awolowo University, Nigeria (6°51′45″ N, 4°79′00″ E) were analyzed (**Figure 1**). The specimens were transferred to laboratory aquaria and kept under standard conditions for 1 day prior to the experiments. As *H. odoe* represent a non-CITES threatened species, no proper authorization was required for their sampling and/or transportation. All specimens were deposited in the Museu de Zoologia of the Universidade de São Paulo (MZUSP), under the accession No. 119844. Mitotic chromosomes were obtained by the protocols described in Bertollo et al. (2015) and experiments followed ethical conducts in accordance with the Ethics Committee on Animal Experimentation of the Universidade Federal de São Carlos (Process number CEUA 1853260315). The C-positive heterochromatin was detected using the Barium hydroxide protocol (Sumner, 1972). The genomic DNA was extracted according to standard phenol–chloroform procedures (Sambrook and Russell, 2001).

### Probes for Chromosome Hybridization

A total of 11 repetitive DNA sequences, including four multigene families (U1 and U2 snDNA, 5S and 18S rDNAs) and seven microsatellite repeat motifs (A)_30_, (CA)_15_, (GA)_15_, (CAC)_10_, (CGG)_10_, (GAA)_10_ and (GAG)_10_, were used as probes for FISH experiments. The oligonucleotide probes were directly labeled with Cy3 during synthesis according to Kubat et al. (2008). The other four tandemly arrayed DNA sequences were obtained via PCR from the nuclear DNA of *H. odoe.* The 5S rDNA repeat copy included 120 base pairs (bp) of the 5S rRNA transcribing gene and 200 bp of the non-transcribed spacer (NTS), produced according to Pendás et al. (1994). The second probe contained 1,400-bp repeats of the 18S rRNA gene, obtained according to Cioffi et al. (2009). Both rDNA probes were cloned into plasmid vectors and propagated in DH5α *Escherichia coli* competent cells (Invitrogen, San Diego, CA, United States). The U1 and U2 snDNA sequences were produced by PCR, according to Cross et al. (2005) and Silva et al. (2015), respectively. All these probes were directly labeled with Spectrum Orange-dUTP by nick translation, according to manufacturer’s recommendations (Roche, Mannheim, Germany), with the exception of 5S rDNA, which was directly labeled with Spectrum Green-dUTP, also by nick translation (Roche, Mannheim, Germany).

### Fluorescence *in Situ* Hybridization (FISH) for Repetitive DNA Mapping

Fluorescence *in situ* hybridization (FISH) was performed under high stringency conditions on metaphase chromosome spreads, as described in Yano et al. (2017a). The chromosome slides were incubated with RNAse (10 μg/mL) for 1 h at 37°C in a wet chamber and then washed for 5 min in 1x PBS and incubated with pepsin 0,005% for 10 min at room temperature. It was followed a wash in 1x PBS, a fixation with 1% formaldehyde for 10 min at room temperature, and another 1x PBS wash. The slides were then set for an alcoholic series of 70, 85, and 100% 2 min each, followed by the DNA denaturation in 70% formamide/2x SSC for 3 min at 75°C. After denaturation, the chromosome spreads were dehydrated in an ethanol series of 70, 85, and 100% at room temperature, 2 min each. 20 μL of the hybridization mixture (100 ng probes, 50% deionized formamide, 10% dextran sulfate) were then dropped on the slides, and the hybridization was performed for 16–18 h at 37°C in a wet chamber containing 2x SSC. A post-hybridization wash was carried out with 2x SSC for 5 min followed by another wash in 1x SSC at 42°C, 5 min. A final washing series was then performed at room temperature, consisting of 1x PBS for 5 min, and ethanol 70, 85, and 100% for 2 min each. Finally, the chromosomes were counterstained with DAPI (1.2 μg/mL) and the slides mounted with an antifading solution (Vector, Burlingame, CA, United States).

### Chromosomal Microdissection, Probe Preparation and Labeling

Fifteen copies of the following chromosomes were isolated by microdissection and amplified using the procedure described in Yang et al. (2009): (i) X chromosome of *Hoplias malabaricus* karyomorph B (HMB-X); (ii) Y_1_ chromosome of *H. malabaricus* karyomorph G (HMG-Y1) and (iii) Y chromosome of *Erythrinus erythrinus* karyomorph D (ERY-Y). These probes were labeled with Spectrum Orange-dUTP (ERY-Y) or Spectrum Green-dUTP (HMB-X and HMG-Y1) (Vysis, Downers Grove, IL, United States) in a secondary DOP PCR using 1 μL of the primarily amplified product as a template DNA, following Yang et al. (2009).

### FISH of Whole Chromosome Specific Probes (W)

Chromosomal preparations of males and females of *H. odoe* were used for Zoo-FISH experiments with all the above mentioned probes. The hybridization procedures followed Yano et al. (2017a). To block the hybridization of high-copy repeat sequences 60 μg of C_0_t-1 DNA directly isolated from *H. malabaricus* (karyomorphs B and G) and *E. erythrinus* (karyomorph D) male genomes were prepared according to Zwick et al. (1997). Hybridization was performed for 144 h at 37°C in a moist chamber. The post-hybridization wash was carried out with 1x SSC for 5 min at 65°C, and in 4x SSC/Tween using a shaker at RT and then rinsed quickly in 1x PBS. Subsequently, the slides were dehydrated in an ethanol series (70, 85, and 100%), 2 min each. Finally, the chromosomes were counterstained with DAPI (1.2 μg/mL) and mounted in antifade solution (Vector).

### Probes for Comparative Genomic Hybridization (CGH)

The gDNA of *H. odoe* was used for comparative analyzes with the gDNAs of several Erythrinidae species, namely *E. erythrinus* (karyomorph D), *Hoplias lacerdae*, *H. malabaricus* (karyomorph A) and *Hoplerythrinus unitaeniatus* (karyomorph D). The gDNA of *H. odoe* was labeled with biotin-16-dUTP using BIO-nick-translation Mix (Roche), while the male-derived gDNAs of *E. erythrinus*, *H. malabaricus*, *H. unitaeniatus*, and *H. lacerdae* were labeled with digoxigenin-11-dUTP using DIG-nick-translation Mix (Roche, Manheim, Germany). In all experiments it was utilized C_0_t-1 DNA (i.e., fraction of genomic DNA enriched for highly and moderately repetitive sequences), prepared according to Zwick et al. (1997), for blocking common genomic repetitive sequences. The final probe was composed of 500 ng of *H. odoe* gDNA plus 500 ng of the corresponding gDNA for each Erythrinidae species. The probe was ethanol-precipitated and the dry pellet dissolved in a hybridization buffer (20 μL per slide) containing 50% formamide + 2x SSC + 10% SDS+ 10% dextran sulfate and Denhardt’s buffer, pH 7.0).

### Fluorescence *in Situ* Hybridization for CGH

CGH experiments were performed according to Symonová et al. (2013). Slides with the metaphase plates were stored overnight in a freezer, being submitted to an alcoholic series of 70, 85, and 100%, 3 min each, before and after the storage. After that, the slides were aged for 1–2 h at 60°C, washed in 2x SSC for 5 min, treated with RNAse (200 μg/mL) for 90 min at 37°C in a wet chamber and them washed in 2x SSC for 30 s. It was followed another alcoholic series treatment, a wash in 1x PBS for 5 min, a Pepsin (50 μg/mL) treatment, a wash in 1x PBS for 5 min and an additional alcoholic series treatment. Finally, the material was denaturated in 75% formamide/2x SSC at 74°C for 3 min, followed by an alcoholic series being the first 70% cold ethanol. 20 μL of the probes were spotted to the slides, which were them incubated at room temperature (37°C) in a dark humid chamber for 3 days, with rubber-sealed coverslips. The rubber cement and coverslips were removed in a solution of 4x SSC/0.1% Tween. The slides were then washed twice in 50% formamide/2x SSC for 10 min each, three times in 1x SSC, rinsed in 2x SSC at room temperature, and incubated 20 min. in a humid chamber with 500 μL of 3%BSA/4x SSC/Tween, with coverslips. The hybridization signal was detected with anti-digoxigenin-Rhodamin (Roche) diluted in 0.5% bovine serum albumin (BSA) in PBS, and avidin-FITC (Sigma) diluted in PBS containing 10% normal goat serum (NGS). Four final washes were performed at 44°C in 4x SSC/0.1% Tween, 7 min each. Finally, the chromosomes were counterstained with DAPI (1.2 μg/mL) and mounted in an antifade solution (Vector, Burlingame, CA, United States).

### Microscopic Analyses

At least 30 metaphase spreads per individual were analyzed to confirm the diploid number, karyotype structure and FISH results. Images were captured using an Olympus BX50 microscope (Olympus Corporation, Ishikawa, Japan) with CoolSNAP and the images processed using Image Pro Plus 4.1 software (Media Cybernetics, Silver Spring, MD, United States). Chromosomes were classified as metacentric (m), submetacentric (sm), subtelocentric (st) or acrocentric (a), according to their arm ratios (Levan et al., 1964).

## Results

### Karyotype Composition and C-banding

**FIGURE 2:**
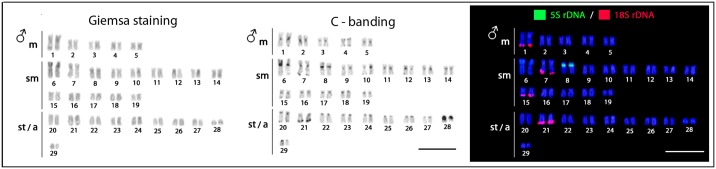
*Hepsetus odoe* male karyotypes under standard Giemsa staining, C-banding and double-FISH with 18S rDNA (red) and 5S rDNA (green) probes. Both males and females have the same karyotypes. Bar = 5 μm.

All specimens, both males and females, have 2n = 58 (10m + 28sm + 20st/a). The C-positive heterochromatic is most localized in the centromeric and subtelomeric regions, with a more conspicuous block present in the 28th chromosome pair of the karyotype (**Figure 2**).

### Chromosomal Mapping of Repetitive DNAs

**FIGURE 3:**
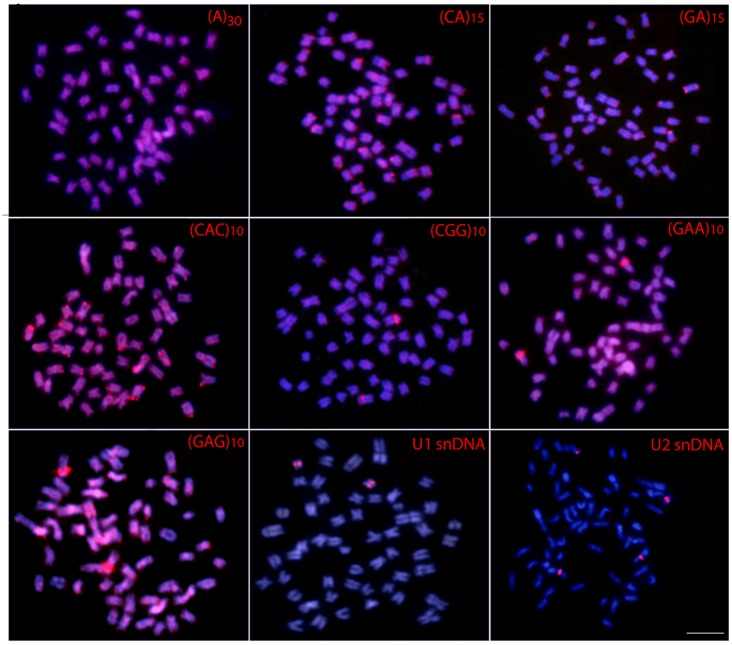
Metaphase plates of *Hepsetus odoe* hybridized with repetitive DNA sequences, including mono-, di- and trinucleotide microsatellites and the multigene families U_1_ and U_2_ snDNAs. Bar = 5 μm.

The 5S rDNA occurs in the proximal region of the short arms of only one sm chromosome pair, while the 18S rDNA is located in the terminal region of the long arms of four chromosome pairs (1m + 2sm + 1st) (**Figure 2**). Clear interstitial hybridization signals were observed in one pair of chromosomes for the U1 snDNA, and in two chromosome pairs for the U2 snDNA, being interstitial and telomeric located in each one of them, respectively (**Figure 3**). Widely distributed marks were evidenced by the microsatellite motifs. Signals were mainly telomeric, but some also interstitial, as for (A)_30_, (CA)_15_, (GA)_15_, (CAC)_10_ and (GAG)_10_ probes. Exceptions for these general patterns were presented by the (CGG)_10_, (GAA)_10_ and (GAG)_10_ probes, which highlighted a conspicuous interstitial signal on a unique pair of chromosomes (**Figure 3**).

### Comparative Genomic Hybridization (CGH)

**FIGURE 4:**
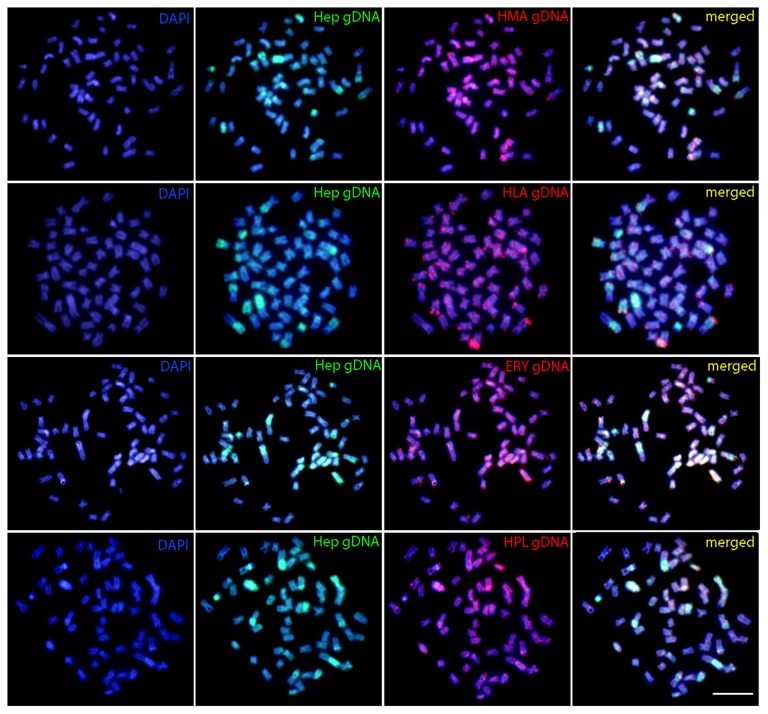
Comparative genomic hybridization (CGH) in metaphase plates of *Hepsetus odoe*. First column: DAPI images (blue); Second column: hybridization pattern with *Hepsetus odoe* (Hep) gDNA probe; Third column: Hybridization patterns with *Hoplias malabaricus* (HMA) gDNA, *Hoplias lacerdae* (HLA) gDNA, *Erythrinus erythrinus* (ERY) gDNA and *Hoplerythrinus unitaeniatus* (HPL) gDNA probes; Fourth column: merged images of each genomic probes and DAPI staining. The common genomic regions are depicted in yellow. Bar = 5 μm.

The comparative genomic hybridization showed that the gDNA of *H. odoe* shares some homologies with those of the Erythrinidae species analyzed. Despite some scattered hybridization, labeled telomeric and pericentromeric regions were evidenced according to each species. However, it stands out the hybridization pattern with *E. erythrinus*, where some whole chromosome pairs were labeled, in addition to telomeric overlaps in other ones. An exclusive acrocentric chromosome of *H. odoe*, that presented hybridization signals only with the gDNA of *H. unitaeniatus*, was also highlighted (**Figure 4**).

### Detection of Chromosomal Homeologies by Zoo-FISH Experiments

**FIGURE 5:**
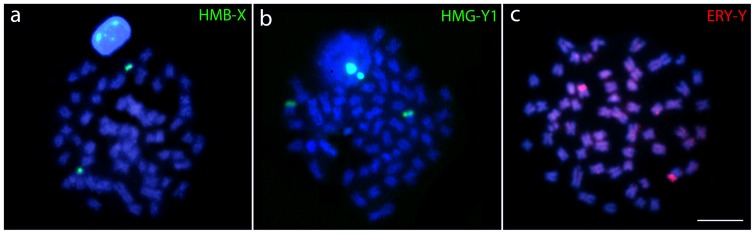
Whole chromosome painting (WCP) in metaphase plates of *Hepsetus odoe* showing the chromosomes hybridized with **(a)** the X chromosome of *Hoplias malabaricus* karyomorph B (HMB-X), **(b)** the Y_1_ chromosome of *Hoplias malabaricus* karyomorph G (HMG-Y_1_) and **(c)** the Y chromosome of *Erythrinus erythrinus* karyomorph D (ERY-Y).

Hybridization performed with HMB-X (X chromosome from *Hoplias malabaricus* karyomorph B) probe highly painted one small st/a chromosome of *H. odoe* (**Figure 5a**). Both HMG-Y1 (Y_1_ chromosome from *H. malabaricus* karyomorph G) and ERY-Y (Y chromosome from *Erythrinus erythrinus* karyomorph D) probes painted the p arms of medium-sized st/a chromosomes (**Figures 5b,c**) of *H. odoe*. Besides, ERY-Y probe also produced faint scattered hybridization pattern on several other chromosomes of *H. odoe* (**Figure 5c**).

## Discussion

### General Chromosome Features of *Hepsetus odoe*

The lack of karyotype data for several fish groups impairs comparative analyzes on their evolutionary trends and chromosomal relationships. This is the case for the African Hepsetidae family for which chromosomal characteristics are completely unknown. In this sense, this study is the first one providing classical and molecular cytogenetic data for one of its representative species, *H. odoe*.

Both male and female specimens of *H. odoe* have the same karyotype structure, with 2n = 58 (5m + 14sm + 10st/a), with no evidence of differentiated sex chromosomes. The heterochromatin distribution follows the general pattern usually found in many other fish species, with preferential centromeric localization. Only one chromosome pair bears proximal 5S rDNA sites in their short arms, in contrast to the 18S rDNA sequences that are located in the telomeric regions of four different pairs in the karyotype. The distribution of these multigene families is shared among many fish groups (Pendás et al., 1994; Gornung, 2013) where the clustering of the 5S and 18S rDNAs in different chromosomes may avoid unwanted chromosomal changes between them (Martins and Galetti, 1999). In addition, this differential clustering is also true for the U2 snDNA sequences, since the cytogenetic mapping for different genes that composed this multigene family, although scarce among fishes, shows a preferential distribution among distinct chromosomes (reviewed in Yano et al., 2017b), as also observed in *H. odoe*.

With respect to microsatellites, although the scattered distribution of some of them is not so useful for comparative approaches, the conspicuous interstitial bands that (GAG)_10_, (CGG)_10_ and (GAA)_10_ probes highlighted in the genome of *H. odoe*, constitute important markers for comparative evolutionary analyzes with other Hepsetidae and also close related species. In fact, the clustering of microsatellites represents important evolutionary stages by composing non-coding genome regions, as well as relevant steps in the sex chromosome’s differentiation process (Bergero and Charlesworth, 2009).

DNA sequence analysis strongly suggests that Hepsetidae and Alestidae are phylogenetic close related families (Oliveira et al., 2011; Arcila et al., 2017). In this sense, the present data set for *H. odoe* are useful tools for complementary investigations covering other Hepsetidae and Alestidae species. In fact, this study represents the first one of a series focusing on the cytogenetics and cytogenomics of such African families, toward the investigation of their karyoevolutionary processes and relatedness.

### Comparative Cytogenetics of *Hepsetus odoe* with Other Characiformes Species

**FIGURE 6:**
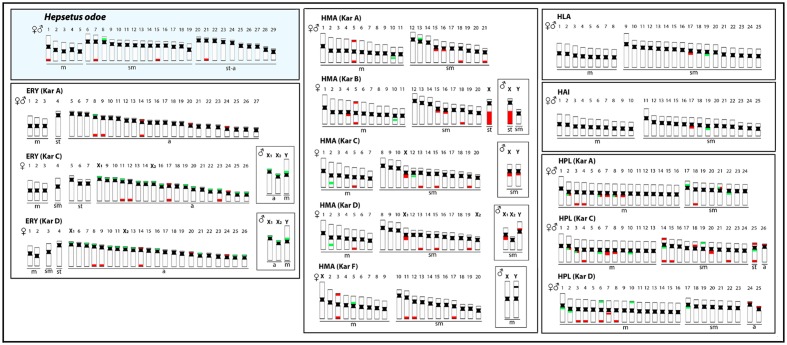
Representative idiograms of *Hepsetus odoe* and Erythrinidae species: *Erythinus erythrinus* (ERY) karyomorphs (Kar) A, C, D; *Hoplias malabaricus* (HMA) karyomorphs A. B, C, D, F; *Hoplias lacerdae* (HLA); *Hoplias aimara* (HAI) and *Hoplerythrinus unitaeniatus* (HPL) karyomorphs A.C, D. The distribution of the 18S and the 5S rDNAs for each species are highlighted in red and green, respectively. The sex chromosomes are boxed. Data from Cioffi et al. (2009), Martins et al. (2013), Martinez et al. (2015), and Oliveira et al. (2015).

**FIGURE 7:**
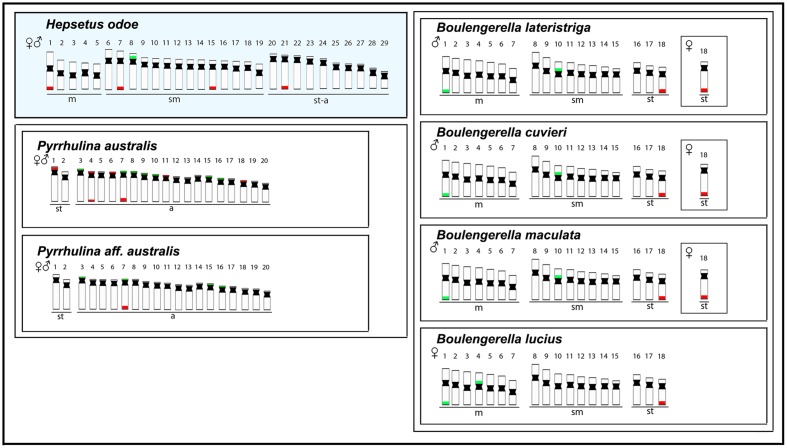
Representative idiograms of *Hepsetus odoe* and *Pyrrhulina* (Lebiasinidae) (data from Moraes et al., 2017) and *Boulengerella* (Ctenoluciidae) (data from de Souza E Sousa et al., 2017) species. The distribution of the 18S and the 5S rDNAs for each species are highlighted in red and green, respectively.

Some previous phylogenetic studies (Ortí and Meyer, 1997; Buckup, 1998; Calcagnotto et al., 2005) have suggested a relationship between Hepsetidae and some other Neotropical groups, such as the Erythrinidae, Ctenoluciidae and Lebiasinidae, although without a full consensus among them. Using new sequencing technology together with phylogenetic reconstructions, a new scenario was evidenced, discarding relationships of Hepsetidae with Lebiasinidae and Cnetoluciidae and, instead off, placing Hepsetidae and Alestidae in a closer clade which has a near position in the phylogenetic tree to some other Neotropical families, such as Erythrinidae, Cynodontidae and Hemiodontidae (Oliveira et al., 2011; Arcila et al., 2017). In this way, as the cytogenetic studies among Cynodontidae and Hemiodontidae families are until now restricted to 2n descriptions in few species, and Alestidae species are still unavailable in spite of recent collecting efforts, we performed a comparative analysis among *H. odoe* and Erythrinidae, Ctenoluciidae and Lebiasinidae representatives. In this sense, **Figures 6**, **7** depict some data, including chromosome number, karyotype organization, sex chromosome systems and distribution of the major and minor rDNA sequences in some Erytrinidae, Lebiasinidae, and Ctneoluciidae species. A general overview clearly indicates that Erythrinidae retains the highest amount of characters resembling those of *H. odoe* than Lebiasinidae and Ctenoluciidae species. Indeed, *Erythrinus erythrinus* (2n = 54/52), *Hoplias lacerdae* and *H. aimara* (2n = 50) and *Hoplerythrinus unitaeniatus* (2n = 48/52) show diploid numbers closer to that of *H. odoe* (2n = 58) then *Pyrrhulina* (2n = 40; Lebiasinadae) and *Boulengerella* (2n = 36; Ctenoluciidae) species.

Particularly, inside Erythrinidae, *E. erythrinus* stand out as having more chromosomal similarities with *H. odoe* than the other ones, taking into account the broad organization of the karyotype and the amount of mono-armed chromosomes that they have. In fact, *E. erythrinus* karyomorph A shows the most basal karyotype inside this genus, considering that the other *Erythrinus* karyomorphs highlight clearly derived features, such as the differentiation of a multiple X_1_X_1_X_2_X_2_/X_1_X_2_Y sex chromosome system (Bertollo et al., 2004) and the huge dispersion of the 5S rDNA sequences in the genome (Cioffi et al., 2010; Martins et al., 2013). In addition, like *H. odoe*, *E. erythrinus* karyomorph A presents only one chromosome pair bearing 5S rDNA sequences at a similar position on the chromosomes, as well as a number of exclusive telomeric 18S rDNA sites. However, whereas in *H. odoe* the major rDNA sequences are only distributed in the long arms of the chromosomes, in *E. erythrinus* they are found both in the short as well as in the long arms (Cioffi et al., 2010). This is not an unexpected condition in view of differential distributions that can be set up along the evolutionary history of the species. In fact, repetitive DNAs have played a particular role on fish karyotyping (Cioffi and Bertollo, 2012), and variations in amount and types of several classes of repetitive DNAs are expected considering the inherent dynamism of these sequences during the evolutionary history of different taxa (Kubat et al., 2008; Cioffi et al., 2010, 2012; Pokorná et al., 2011; Yano et al., 2016). In spite of this, the distribution pattern of the (GAG)_10_ microsatellites in *H. odoe* also shows a significant accumulation on the *E. erythrinus* chromosomes (Yano et al., 2014).

Considering the above correlations between *Hepsetus* and Erythrinidae, comparative genomic hybridization (CGH) and whole chromosome painting (WCP) were also performed to obtain additional informative markers for comparative cytogenetics. Among fishes, CGH has been already applied for several purposes, such as to compare genomes of closely related species (Zhu and Gui, 2007; Knytl et al., 2013; Majtánová et al., 2016; Moraes et al., 2017), to detect parental genomes in hybrids (Symonová et al., 2013; Pereira et al., 2014), and to elucidate the origin and evolution of B and sex chromosomes (Fantinatti et al., 2011; Freitas et al., 2017; Yano et al., 2017c), among others. In our present case, CGH with four Erythrinidae species evidenced the co-localization of scattered signals in almost all chromosomes of *H. odoe*, together with the preferential signals in the terminal parts of some chromosomes, thus indicating the shared repetitive content of such regions. However, it stands out the hybridization pattern with *E. erythrinus*, where some whole chromosome pairs were painted, in addition to telomeric overlaps in other ones. Furthermore, the hybridization with *H. odoe* gDNA revealed the occurrence of conspicuous species-specific regions, very likely as a result of its particular evolutionary history, given that the resolution of the CGH method predominantly relies on the presence of species-specific (or sex-specific) repetitive DNA sequences and the evolutionary distance of the compared genomes.

Besides CGH, WCP experiments were also performed using microdissected sex chromosomes from *H. malabaricus* karyomorphs B (HMB-X) and G (HMG-Y_1_) and *E. erythrinus* karyomorph D (ERY-Y) as probes, in order to verify the occurrence of putative sex chromosomes in *H. odoe*. As a control experiment, all probes were previously hybridized in male chromosomal preparations of *H. malabaricus* (karyomorphs B and G) and *E. erythrinus* (karyomorph D), clearly demonstrating the hybridization signals on the sex chromosomes of these karyomorphs, thus corroborating previous data (Cioffi et al., 2013; Oliveira et al., 2017). When these probes were hybridized to chromosomal preparations of *H. odoe*, HMB-X highly painted one small st/a chromosome, while HMG-Y1 and ERY-Y probes painted the p arms of medium-sized st/a chromosomes. This way, these results highlight that such linkage groups are shared by *H. odoe* and Erythrinidae species, corroborating the CGH experiments which also demonstrated the sharing of a considerable genomic fraction among such groups. The maintenance of such linkage groups is somehow surprising considering the phylogenetic distance between these clades. However, chromosome homology across widely phylogenetically distributed clades have been also detected in several mammals (Balmus et al., 2007; Dementyeva et al., 2010; Kulemzina et al., 2011), birds (Oliveira et al., 2008, 2010; Tagliarini et al., 2011) and lizard (Pokorná et al., 2011) species. In the later, Zoo-FISH experiments using a Z-derived probe from *Gallus gallus* showed that the fraction of the reptile genome that is homologous to the avian Z chromosome exhibits a conserved synteny, despite the very ancient times (∼275 Mya) of their divergence (Pokorná et al., 2011).

## Conclusion

This study, focusing on standard and molecular cytogenetic approaches of *H. odoe*, represents the first data set for an Hepsetidae species. Our data supports the likely proximity between African and Neotropical families, such as Hepsetidae and Erythrinidae. In fact, our experiments, including CGH and WCP, indicate that *H. odoe* and some Erythrinidae species, in special from the genus *Erythrinus*, share similar chromosomal sequences, thus reflecting some degree of relationship among them. In fact, *Erythrinus* seems to carry the most basal karyotype organization within Erythrinidae, and likely the most proximal to that highlighted by *H. odoe*. This study represents the first one of a series of further investigations focusing on the African Characiformes chromosomal and genomic characteristics, allowing a broader and more detailed view on the evolutionary history of this group through a cytogenetic approach. Such additional data will securely improve our knowledge about the relatedness of the African and the Neotropical characiform families.

## Author Contributions

PC carried out the cytogenetic analysis and drafted the manuscript. EdO, CY, AA-R, and TH helped in the cytogenetic analysis, drafted and revised the manuscript. CO, ED, OJ, and TL drafted and revised the manuscript. MC and LB coordinated the study, drafted and revised the manuscript. All authors read and approved the final version of the manuscript.

## Conflict of Interest Statement

The authors declare that the research was conducted in the absence of any commercial or financial relationships that could be construed as a potential conflict of interest.
